# Association of Stress-Induced Hyperglycemia and Diabetic Hyperglycemia with Mortality in Patients with Traumatic Brain Injury: Analysis of a Propensity Score-Matched Population

**DOI:** 10.3390/ijerph17124266

**Published:** 2020-06-15

**Authors:** Yu-Chin Tsai, Shao-Chun Wu, Ting-Min Hsieh, Hang-Tsung Liu, Chun-Ying Huang, Sheng-En Chou, Wei-Ti Su, Shiun-Yuan Hsu, Ching-Hua Hsieh

**Affiliations:** 1 Department of Neurosurgery, Kaohsiung Chang Gung Memorial Hospital, Chang Gung University College of Medicine, Kaohsiung 83301, Taiwan; vagility@gmail.com; 2 Department of Anesthesiology, Kaohsiung Chang Gung Memorial Hospital, Chang Gung University College of Medicine, Kaohsiung 83301, Taiwan; shaochunwu@gmail.com; 3 Department of Trauma Surgery, Kaohsiung Chang Gung Memorial Hospital, Chang Gung University College of Medicine, Kaohsiung 83301, Taiwan; hs168hs168@gmail.com (T.-M.H.); htl1688@yahoo.com.tw (H.-T.L.); junyinhaung@yahoo.com.tw (C.-Y.H.); athenechou@gmail.com (S.-E.C.); s101132@adm.cgmh.org.tw (W.-T.S.); ah.lucy@hotmail.com (S.-Y.H.); 4 Department of Plastic Surgery, Kaohsiung Chang Gung Memorial Hospital, Chang Gung University College of Medicine, Kaohsiung 83301, Taiwan

**Keywords:** stress-induced hyperglycemia, diabetic hyperglycemia, traumatic brain injury, mortality

## Abstract

**Background:** Hyperglycemia at the time of hospital admission is associated with higher morbidity and mortality rates in patients with traumatic brain injury (TBI). Using data from the Chang Gung Research Database (CGRD), this study aimed to compare mortality outcomes between patients with stress-induced hyperglycemia (SIH), diabetic hyperglycemia (DH), and nondiabetic normoglycemia (NDN). The study occurred at Keelung, Linkou, Chiayi, and Kaohsiung Chang Gung Memorial Hospitals (CGMHs). **Methods:** A total of 1166, 6318, 3622, and 5599 health records from Keelung, Linkou, Chiayi, and Kaohsiung CGMHs, respectively, were retrieved from the CGRD for hospitalized patients with TBI between January 2001 and December 2015. After propensity score matching for sex, age, and Glasgow Coma Scale (GCS) score, the matched cohorts were compared to evaluate differences in the primary outcome between patients with SIH, DH, and NDN. In-hospital mortality was the primary outcome. **Results:** The analysis of matched patient populations revealed that at the Kaohsiung CGMH, patients with SIH had 1.63-fold (95% CI: 1.09–2.44; *p* = 0.017) and 1.91-fold (95% CI: 1.12–3.23; *p* = 0.017) higher odds of mortality than patients with NDN and DH, respectively. Similar patterns were found at the Linkou CGMH; patients with SIH had higher odds of mortality than patients with NDN and DH. In contrast, at the Keelung CGMH, patients with SIH had significantly higher odds of mortality than those with NDN (OR: 3.25; 95% CI: 1.06–9.97; *p* = 0.039). At the Chiayi CGMH, there were no significant differences in mortality rates among all groups. **Conclusions:** This study’s results suggest that SIH and DH differ in their effect on the outcomes of patients with TBI. The results were similar between medical centers but not nonmedical centers; in the medical centers, patients with SIH had significantly higher odds of mortality than patients with either NDN or DH.

## 1. Introduction

Following traumatic brain injury (TBI), hyperglycemia may contribute to lactic acidosis in brain tissue, subsequently resulting in neuronal injury [1,2]. Hyperglycemia is associated with higher morbidity and mortality in patients [3,4]. Hyperglycemia observed at the time as hospital admission may be either diabetic hyperglycemia (DH) or stress-induced hyperglycemia (SIH). DH and SIH are diagnosed when serum glucose concentrations are ≥200 mg/dL in patients with and without diabetes mellitus (DM), respectively [5]. A stress response can commonly induce a form of SIH in patients with critical illnesses such as TBI [6,7,8], myocardial infarction [9], femoral fracture [10], or major trauma [11,12,13,14]. Unlike DH, which is a chronic process associated with microvascular changes in cases of prolonged hyperglycemia [15], SIH occurs secondary to increased hepatic output of glucose. SIH is also characterized by diminished insulin production and insulin resistance in peripheral tissues; furthermore, those with SIH exhibit excessive adrenal cortical output and high levels of circulating inflammatory cytokines [15,16]. 

There is an association between SIH and increased mortality following TBI [7,15]. However, there is limited information regarding whether patients with SIH and TBI represent a distinct group with outcomes differing from those of patients with DH. Bosarge et al. (2015) reported that after adjusting for age, sex, injury severity, and lactic acid levels, patients with SIH and severe TBI had a 50% higher mortality rate than patients with severe TBI and nondiabetic normoglycemia (NDN) [7]. In contrast, patients with severe TBI and DH did not have a significantly higher mortality rate than those with severe TBI and NDN [7]. Our prior study regarding patients with isolated moderate-to-severe TBI [16], which was performed in a level I trauma center, revealed that patients with SIH and DH had 9.1-fold and 2.3-fold higher odds of mortality, respectively, than those with NDN. After propensity score matching for sex, age, preexisting comorbidities, the presence of different intracerebral hemorrhages, and injury severity, patients with SIH still had 6.6-fold higher odds of mortality than those with NDN. However, patients with DH did not show significantly higher mortality odds than those with NDN after adjusting for the aforementioned variables. 

Given that our previous study was limited to a single urban Level I trauma center, we were interested in whether such observations would be detected in other regions. Therefore, in this study, we aimed to compare the mortality outcomes between patients with TBI and either SIH, DH, or NDN using the Chang Gung Research Database (CGRD), which comprises medical data from the largest private hospital system in Taiwan, namely, the Chang Gung Memorial Hospital (CGMH). The CGRD encompasses 6.1% of the outpatients and 10.2% of the hospitalized patients in Taiwan [17]. The hypothesis of this study was that, regardless of the level of hospital (medical center or nonmedical center), the associated odds of mortality in patients with TBI and SIH, DH, or NDN would not be significantly different. 

## 2. Materials and Methods

### 2.1. Ethics Statement

This cross-sectional retrospective study used registered data in the trauma registry system of the CGMH. This study was approved by the Institutional Review Board (IRB) of the CGMH (approval number 201600533B0). Informed consent was waived according to IRB regulations.

### 2.2. Inclusion Criteria for Patient Groups: Diagnostic Methods

Hyperglycemia was defined as serum glucose concentrations of ≥200 mg/dL in the emergency department. Using the American Diabetes Association’s current recommendations for DM diagnosis, DM was diagnosed by patient history (International Classification of Diseases, Ninth Revision [ICD-9] code 250) and/or glycated hemoglobin (HbA1c) levels of ≥6.5% on admission [5]. NDN was defined as serum glucose concentrations of <200 mg/dL in patients without DM. Diabetic normoglycemia (DN) was defined as serum glucose concentrations of <200 mg/dL in patients with DM. DH and SIH were diagnosed when serum glucose concentrations were ≥200 mg/dL in patients with and without DM, respectively. In the present study, enrolled patients from each hospital were allocated into four mutually exclusive groups (NDN, DN, SIH, DH) based on the above criteria.

### 2.3. Study Population

**Figure 1:**
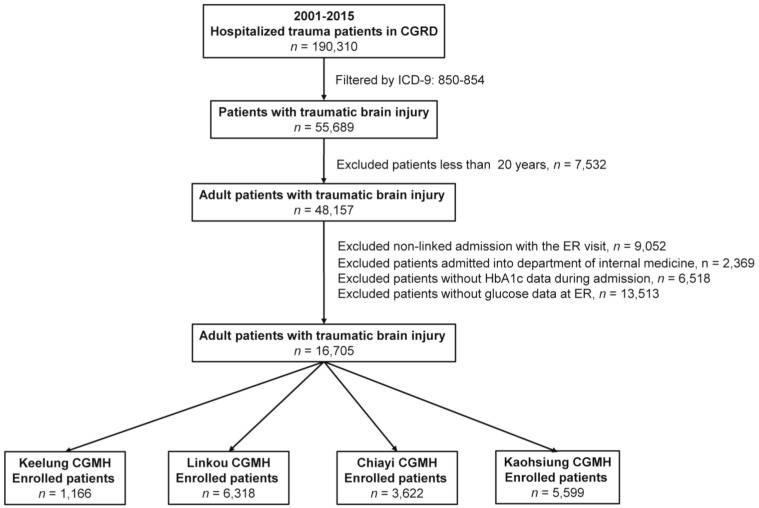
Flow chart illustrating the inclusion and exclusion of adult patients with Table 2.

Only patients with available data on serum glucose levels in the emergency department and patients with a history of DM or available HbA1c level data within three months on admission were included. Patients with incomplete data were excluded from the study. As shown in Figure 1, the medical data and original medical records of the 190,310 hospitalized trauma patients in the CGRD from January 2001 to December 2015 were obtained from four medical institutes: the Keelung, Linkou, Chiayi, and Kaohsiung CGMHs, which are located in the northeast, northern, central, and southern regions of Taiwan, respectively. The Linkou and Kaohsiung CGMHs are urban medical centers with a Level I trauma center. The extraction of patient information under ICD-9 codes 850 to 854 (850: concussion; 851: cerebral laceration and contusion; 852: subarachnoid, subdural, and extradural hemorrhage following injury; 853: other and unspecified intracranial hemorrhage following injury; 854: intracranial injury of other and unspecified nature) resulted in 55,689 patients with TBI being included in the study. We further excluded patients under 20 years of age (*n* = 7532). Additionally, individuals were excluded if their admission could not be linked with an emergency room visit (*n* = 9052) or if they were admitted to an internal medicine department (*n* = 2369). Patients without HbA1c level data during that particular admission (*n* = 6518) and those without serum glucose level data at the emergency department (*n* = 13,513) were also excluded. Following exclusions, 16,705 adult patients with TBI were included in this study. This included 1166, 6318, 3622, and 5599 patients in the Keelung, Linkou, Chiayi, and Kaohsiung CGMHs, respectively (Figure 1). These patients’ medical information, including data on sex, age, Glasgow Coma Scale (GCS) score, and status at discharge, including mortality or survival, was retrieved from the database. 

### 2.4. Statistical Analysis

Statistical analyses were performed using SPSS 23.0 software for Windows (IBM Corp., Armonk, NY, USA). In each hospital (Keelung, Linkou, Chiayi, Kaohsiung), comparisons were made among the four patient groups (NDN, DN, SIH, DH). The continuous variables were analyzed using one-way analysis of variance following Games-Howell post-hoc tests and were expressed as mean ± standard deviation. Two-sided Fisher’s exact or Pearson’s chi-squared (χ^2^) tests were used to compare categorical data, with odds ratios (ORs) being calculated with 95% confidence intervals (CIs). To minimize the confounding effects of baseline characteristics between patient populations when assessing mortality outcomes, a 1:1 propensity score-matched study group was created by the Greedy method with a 0.2 caliper width using NCSS 10 software (NCSS Statistical Software, Kaysville, Utah). The propensity scores were calculated using a logistic regression model, including the following covariates: sex, age, and GCS score. After adjusting for these confounding factors, Cox regression was used to evaluate the effects of SIH and DH on the primary outcome compared to NDN. Additionally, this method was used to examine differences between the SIH and DH patient groups. The primary outcome measure was in-hospital mortality. A *p* value of < 0.05 was set to determine statistically significant group differences. 

## 3. Results

### 3.1. Demographics and Patient Outcomes

The patients enrolled in this study were categorized into four groups: NDN, DN, SIH, and DH. The mortality rate ranged from 2.1% to 5.4% for patients with NDN, 10.8% to 21.2% for patients with SIH, and 3.6% to 10.7% for patients with DH (Table 1). The GSC scores of patients with SIH were significantly lower at the Linkou (8.2 ± 4.6) and Kaohsiung (8.5 ± 4.9) CGMHs than at the Keelung (11.0 ± 4.6) and Chiayi (10.5 ± 5.0) CGMHs (all *p* < 0.001). As shown in Table 2, at the Kaohsiung CGMH, patients with SIH and DH had 5.81-fold (95% CI: 4.40–7.68; *p* < 0.001) and 2.15-fold (95% CI: 1.48–3.12; *p* < 0.001) higher odds of mortality, respectively, than patients with NDN. Additionally, patients with SIH had 2.71-fold (95% CI: 1.80–4.08; *p* < 0.001) higher odds of mortality than patients with DH. Similar patterns were observed at the Linkou CGMH, as patients with SIH and DH had 4.68-fold (95% CI: 3.80–5.78; *p* < 0.001) and 1.68-fold (95% CI: 1.21–2.35; *p* = 0.002) higher odds of mortality, respectively, than patients with NDN. Furthermore, patients with SIH had 2.78-fold (95% CI: 1.96–3.96; *p* < 0.001) higher odds of mortality than patients with DH. In contrast, at the Keelung CGMH, higher odds of mortality were only found in patients with SIH (OR: 4.90; 95% CI: 2.76–8.70; *p* < 0.001), not in patients with DH, when compared to patients with NDN. There were no significant differences in mortality between patients with SIH and DH. At the Chiayi CGMH, higher odds of mortality were only found in patients with SIH (OR: 5.71; 95% CI: 3.67–8.89; *p* < 0.001), not in patients with DH, when compared to patients with NDN. However, patients with SIH had 3.29-fold (95% CI: 1.54–7.01; *p* = 0.001) higher odds of mortality than patients with DH. In other words, patients with SIH had significantly higher mortality odds than patients with NDN in all four hospitals, and patients with DH had significantly higher mortality odds than patients with NDN at the Linkou and Kaohsiung CGMHs but not at the Keelung and Chiayi CGMHs. Additionally, patients with SIH showed significantly higher mortality odds than patients with DH at the Linkou, Chiayi, and Kaohsiung CGMHs but not at the Keelung CGMH.

### 3.2. Outcomes of the Matched Patients

To control for the confounding effects of sex, age, and GCS score in the compared patient populations on outcomes, several well-balanced 1:1 propensity score-matched study groups were created for comparisons of patients with DN, SIH, and DH vs. patients with NDN as well as patients with SIH vs. patients with DH in each hospital (Table 3). Among these selected well-balanced pairs of patients, there were no significant differences in sex, age, and GCS scores across the two selected patient cohorts. Analysis of these matched patient populations (Table 4) revealed that, at the Kaohsiung CGMH, patients with SIH had 1.63-fold (95% CI: 1.09–2.44; *p* = 0.017) and 1.91-fold (95% CI: 1.12–3.23; *p* = 0.017) higher odds of mortality than patients with NDN and DH, respectively. A similar pattern was found at the Linkou CGMH, as patients with SIH had 2.08-fold (95% CI: 1.53–2.83; *p* < 0.001) and 2.82-fold (95% CI: 1.73–4.58; *p* < 0.001) higher odds of mortality than patients with NDN and DH, respectively. However, at the Keelung CGMH, patients with SIH had significantly higher odds of mortality than those with NDN (OR: 3.25; 95% CI: 1.06–9.97; *p* = 0.039); however, there were no significant differences in mortality rates between patients with SIH and DH (OR: 1.25; 95% CI: 0.34–4.66; *p* = 0.739). At the Chiayi CGMH, there were no significant differences in mortality rates among the four groups of patients. Analysis of mortality outcomes in the matched patients from all four hospitals revealed that patients with SIH showed significantly higher odds of mortality than patients with NDN at the Keelung, Linkou, and Kaohsiung CGMHs but not at the Chiayi CGMH. Higher odds of mortality were not observed in patients with DH in comparison to patients with NDN across all four hospitals. Furthermore, patients with SIH showed significantly higher odds of mortality than patients with DH at the Linkou and Kaohsiung CGMHs but not at the Keelung and Chiayi CGMHs.

## 4. Discussion

This study was performed across four hospitals, which consisted of two medical centers and two nonmedical centers of the same hospital system. The results reveal that patients with SIH showed significantly higher odds of mortality than patients with NDN in three of the four hospitals. Additionally, patients with DH did not show significantly higher odds of mortality than patients with NDN across all four hospitals. Furthermore, the patterns were similar across the two medical centers (Linkou and Kaohsiung) in comparison with the two nonmedical centers, as patients with SIH showed significantly higher odds of mortality than patients with either NDN or DH.

The results of this study suggest that SIH and DH differ in their effect on patient outcomes after TBI. Although the mechanism underlying the detrimental effects of these two hyperglycemia states is unknown, the pathophysiological effects associated with SIH might differ from those of DH. However, it remains unknown as to why different patterns of mortality risk exist between the patients from medical centers in comparison to nonmedical centers. The potential reasons for this observation remain to be explored. Such discrepancies in risk may be attributed to different patient characteristics in the local regions served by the hospitals or the different interventions provided by the hospitals. Reasonably, the patient populations may differ in injury severity between the medical centers and nonmedical centers. As observed in this study, the GSC scores of the patients with SIH were significantly lower in the Linkou and Kaohsiung CGMHs than in the Keelung and Chiayi CGMHs. Our previous study [14] was performed with data from the trauma registry database in our hospital [18,19,20], where the matched patients could be adjusted not only by patient characteristics, but also by injury severity score (ISS). However, in the current study, the matched patient population could only be adjusted for GCS score. Although GCS scores may partly reflect injury severity, these two patient groups were quite different, as GCS score only indicates brain injury severity, while ISS reflects the sum of injuries over the patients’ six body regions. Unavailable data regarding the patients’ injury severity or abbreviated injury scale (AIS) scores of each body region may have led to bias in the outcome measurement because some patients may have passed away due to body injury rather than brain injury. Although the results of this study are derived from data from the same hospital system, the results from the Kaohsiung CGMH were similar to the results of our previous study [14], which indicated that patients with SIH, but not DH, showed higher mortality odds than patients with NDN. Additionally, patients with SIH had higher odds of mortality than patients with DH. The unknown condition of the patients’ injury and the impossibility of excluding patients with polytrauma in the present study are limitations to consider when interpreting the results of this study. 

Taiwan is a heavily populated island of 36,188 km^2^, with 23 million people living there. There are 19 medical centers across the island. The medical centers are accredited by the appraisal system of the Ministry of Health and Welfare every four years. Most medical resources are unequally distributed in Taiwan; the top 10 medical centers are all located in urban areas and consume a quarter of the national health expenditure, while the medical resources and medical manpower are under-distributed in rural areas [21]. However, it is hard to determine whether the discrepancy in the findings between the medical and nonmedical centers in this study was due to the different interventions provided by the medical or nonmedical centers or due to the different characteristics of the patients served by the medical or nonmedical centers in different regions. This study implies that the comparison of the outcomes of patients with SIH or DH in the evaluation of various intervention procedures may need to be limited to the same level of hospital. Furthermore, a prospective study with a controlled study population may provide more valuable information for the outcome measurement. Although these four hospitals have accredited trauma surgeons and neurosurgeons trained within the same hospital system, unknown factors, such as management guidelines, resuscitation statuses, critical care resources, surgical intervention indications, and the use of glycemic control measures, may have contributed to bias in the outcome measurement (i.e., mortality odds). In a study of 7404 intensive care adult patients with type 2 diabetes mellitus, preadmission metformin use as monotherapy or in combination with other antidiabetic drugs was associated with reduced 30-day mortality [22]. In this study, the patients with DN may have used antidiabetic drugs; however, the use of such drugs was unknown in this study and thus may have led to bias in the assessment of mortality outcomes. 

The present study had several other limitations. The retrospective study design could have led to a selection bias. In addition, only in-hospital mortality was measured, and data on long-term mortality were not included; thereby, the results do not reflect the full scope of mortality. Moreover, because some degree of the stress response could have invoked hyperglycemia in patients with DH, the SIH and DH groups are not mutually exclusive. Moreover, we were unable to measure stress response hormone levels or catecholamine levels, resulting in an inability to specifically identify whether stress might be more responsible for the hyperglycemia in the patients with DH. Furthermore, the potential inaccuracy of the ICD-9 codes is a limitation of this study. Finally, although the CGRD could serve as the basis for accurate mortality estimates in medical studies [17,23,24], the lack of validation of the registered data may be a limitation in the interpretation of results derived from this database. 

## 5. Conclusions

The results of this study suggest that SIH and DH differ in their effect on the outcomes of patients following TBI. Additionally, the results were similar across the medical centers in comparison with the nonmedical centers, as patients with SIH showed significantly higher odds of mortality than patients with either NDN or DH.

## Figures and Tables

**Table 1:** Demographics and outcomes of the enrolled patients with traumatic brain injuries in each hospital categorized into four groups (NDN, DN, SIH, DH).

Keelung CGMH	NDN (*n* = 950)	DN (*n* = 56)	SIH (*n* = 104)	DH (*n* = 56)
Female, *n* (%)	319(33.6)	23(41.1)	41(39.4)	22(39.3)
Age (years)	49.6 ± 20.3	72.8 ± 9.1	51.7 ± 19.4	62.4 ± 14.1
GCS	13.9 ± 2.8	13.9 ± 2.8	11.0 ± 4.6	12.3 ± 3.8
Mortality, *n* (%)	44(4.6)	2(3.6)	20(19.2)	6(10.7)
Linkou CGMH	NDN (*n* = 4729)	DN (*n* = 279)	SIH (*n* = 811)	DH (*n* = 499)
Female, *n* (%)	1276(27.0)	102(36.6)	262(32.3)	179(35.9)
Age (years)	47.9 ± 20.1	69.4 ± 12.4	49.7 ± 20.4	64.8 ± 13.4
GCS	11.8 ± 4.2	12.3 ± 3.8	8.2 ± 4.6	11.0 ± 4.6
Mortality, *n* (%)	257(5.4)	15(5.4)	172(21.2)	44(8.8)
Chiayi CGMH	NDN (*n* = 2884)	DN (*n* = 180)	SIH (*n* = 305)	DH (*n* = 253)
Female, *n* (%)	1022(35.4)	63(35.0)	104(34.1)	91(36.0)
Age (years)	51.6 ± 20.5	68.0 ± 12.0	53.8 ± 19.8	64.5 ± 12.3
GCS	13.7 ± 2.9	13.9 ± 2.7	10.5 ± 5.0	13.5 ± 3.1
Mortality, *n* (%)	60(2.1)	5(2.8)	33(10.8)	9(3.6)
Kaohsiung CGMH	NDN (*n* = 4275)	DN (*n* = 352)	SIH (*n* = 496)	DH (*n* = 476)
Female, *n* (%)	1412(33.0)	154(43.8)	165(33.3)	202(42.4)
Age (years)	48.4 ± 20.1	68.3 ± 11.3	50.2 ± 19.6	62.5 ± 12.9
GCS	12.7 ± 3.7	13.3 ± 3.2	8.5 ± 4.9	12.1 ± 4.1
Mortality, *n* (%)	157(3.7)	12(3.4)	90(18.1)	36(7.6)

CGMH = Chang Gung Memorial Hospital; DH = diabetic hyperglycemia; DN = diabetic normoglycemia; GCS = Glasgow coma scale; NDN = nondiabetic normoglycemia; SIH = stress-induced hyperglycemia.

**Table 2:** Comparison among the four groups (NDN, DN, SIH, DH) of patients with traumatic brain injuries within each hospital.

	DN vs. NDN		SIH vs. NDN		DH vs. NDN		SIH vs. DH	
	OR (95% CI)	*p*	OR (95% CI)	*p*	OR (95% CI)	*p*	OR (95% CI)	*p*
Keelung CGMH								
Female	1.38 (0.80–2.39)	0.250	1.29 (0.85–1.95)	0.233	1.28 (0.74–2.26)	0.381	1.01 (0.52–1.96)	0.986
Age	―	<0.001	―	0.301	―	<0.001	―	<0.001
GCS	―	0.855	―	<0.001	―	0.005	―	0.064
Mortality	0.76 (0.18–3.23)	1.000	4.90 (2.76–8.70)	<0.001	2.47 (1.01–6.07)	0.053	1.98 (0.75–5.27)	0.164
Linkou CGMH								
Female	1.56 (1.21–2.01)	0.001	1.29 (1.10–1.52)	0.002	1.51 (1.25–1.84)	<0.001	0.85 (0.68–1.08)	0.185
Age	―	<0.001	―	0.017	―	<0.001	―	<0.001
GCS	―	0.040	―	<0.001	―	<0.001	―	<0.001
Mortality	0.99(0.58-1.69)	0.967	4.68 (3.80–5.78)	<0.001	1.68 (1.21–2.35)	0.002	2.78 (1.96–3.96)	<0.001
Chiayi CGMH								
Female	0.98 (0.72–1.35)	0.905	0.94 (0.74–1.21)	0.642	1.02 (0.78–1.34)	0.865	0.92 (0.65–1.31)	0.645
Age	―	<0.001	―	0.067	―	<0.001	―	<0.001
GCS	―	0.158	―	<0.001	―	0.326	―	<0.001
Mortality	1.35 (0.53–3.39)	0.430	5.71 (3.67–8.89)	<0.001	1.74 (0.85–3.54)	0.125	3.29 (1.54–7.01)	0.001
Kaohsiung CGMH								
Female	1.58 (1.27–1.97)	<0.001	1.01 (0.83–1.23)	0.915	1.50 (1.23–1.81)	<0.001	0.68 (0.52–0.88)	0.003
Age	―	<0.001	―	0.059	―	<0.001	―	<0.001
GCS	―	0.002	―	<0.001	―	<0.001	―	<0.001
Mortality	0.93 (0.51–1.68)	0.800	5.81 (4.40–7.68)	<0.001	2.15 (1.48–3.12)	<0.001	2.71 (1.80–4.08)	<0.001

CGMH = Chang Gung Memorial Hospital; DH = diabetic hyperglycemia; DN = diabetic normoglycemia; GCS = Glasgow coma scale; NDN = nondiabetic normoglycemia; SIH = stress-induced hyperglycemia.

**Table 3:** Well-balanced propensity score-matched cohorts among the four groups (NDN, DN, SIH, DH) of patients with traumatic brain injuries within each hospital were created.

Propensity Score–Matched Cohorts					
Keelung CGMH					
DN vs. NDN	DN (*n* = 56)	NDN (*n* = 56)	OR (95% CI)	*p*	SD
Sex			1.00 (0.47–2.12)	1.000	0.00%
Male	33(58.9)	33(58.9)			
Female	23(41.1)	23(41.1)			
Age	72.8 ± 9.1	73.0 ± 9.1	―	0.942	−0.68%
GCS	13.9 ± 2.8	14.1 ± 2.4	―	0.668	−3.42%
SIH vs. NDN	SIH (*n* = 102)	NDN (*n* = 102)	OR (95% CI)	*p*	SD
Sex			1.00 (0.57–1.75)	1.000	0.00%
Male	62(60.8)	62(60.8)			
Female	40(39.2)	40(39.2)			
Age	51.4 ± 19.4	50.8 ± 18.5	―	0.831	1.38%
GCS	11.2 ± 4.5	11.2 ± 4.6	―	0.939	−0.72%
DH vs. NDN	DH (*n* = 53)	NDN (*n* = 53)	OR (95% CI)	*p*	SD
Sex			1.00 (0.45–2.21)	1.000	0.00%
Male	34(64.2)	34(64.2)			
Female	19(35.8)	19(35.8)			
Age	61.5 ± 13.8	62.3 ± 14.8	―	0.766	−3.84%
GCS	12.8 ± 3.3	13.0 ± 3.1	―	0.760	−3.92%
SIH vs. DH	SIH (*n* = 47)	DH (*n* = 47)	OR (95% CI)	*p*	SD
Sex			1.00 (0.43–2.32)	1.000	0.00%
Male	30(63.8)	30(63.8)			
Female	17(36.2)	17(36.2)			
Age	61.1 ± 14.3	60.7 ± 13.3	―	0.870	1.23%
GCS	12.0 ± 4.1	12.2 ± 3.8	―	0.797	−3.02%
Linkou CGMH					
DN vs. NDN	DN (*n* = 279)	NDN (*n* = 279)	OR (95% CI)	*p*	SD
Sex			1.00 (0.71–1.41)	1.000	0.00%
Male	177(63.4)	177(63.4)			
Female	102(36.6)	102(36.6)			
Age	69.4 ± 12.4	69.3 ± 12.5	―	0.970	0.24%
GCS	12.3 ± 3.8	12.3 ± 3.8	―	0.956	0.44%
SIH vs. NDN	SIH (*n* = 810)	NDN (*n* = 810)	OR (95% CI)	*p*	SD
Sex			1.00 (0.81–1.23)	1.000	0.00%
Male	549(67.8)	549(67.8)			
Female	261(32.2)	261(32.2)			
Age	49.7 ± 20.3	49.9 ± 20.0	―	0.868	−1.42%
GCS	8.3 ± 4.6	8.3 ± 4.6	―	0.961	0.12%
DH vs. NDN	DH (*n* = 499)	NDN (*n* = 499)	OR (95% CI)	*p*	SD
Sex			1.00 (0.77–1.30)	1.000	0.00%
Male	320(64.1)	320(64.1)			
Female	179(35.9)	179(35.9)			
Age	64.8 ± 13.4	64.8 ± 13.4	―	0.964	0.30%
GCS	11.0 ± 4.6	11.0 ± 4.6	―	0.972	0.26%
SIH vs. DH	SIH (*n* = 399)	DH (*n* = 399)	OR (95% CI)	*p*	SD
Sex			1.00 (0.74–1.34)	1.000	0.00%
Male	268(67.2)	268(67.2)			
Female	131(32.8)	131(32.8)			
Age	63.4 ± 14.0	62.7 ± 13.5	―	0.513	4.82%
GCS	9.9 ± 4.7	10.2 ± 4.7	―	0.308	−5.67%
Chiayi CGMH					
DN vs. NDN	DN (*n* = 180)	NDN (*n* = 180)	OR (95% CI)	*p*	SD
Sex			1.00 (0.65–1.54)	1.000	0.00%
Male	117(65.0)	117(65.0)			
Female	63(35.0)	63(35.0)			
Age	68.0 ± 12.0	68.0 ± 12.0	―	0.993	0.07%
GCS	13.9 ± 2.7	13.9 ± 2.7	―	0.984	0.12%
SIH vs. NDN	SIH (*n* = 304)	NDN (*n* = 304)	OR (95% CI)	*p*	SD
Sex			1.00 (0.72–1.40)	1.000	0.00%
Male	201(66.1)	201(66.1)			
Female	103(33.9)	103(33.9)			
Age	53.7 ± 19.8	54.0 ± 18.6	―	0.868	−1.32%
GCS	10.5 ± 4.9	10.6 ± 4.9	―	0.934	−0.71%
DH vs. NDN	DH (*n* = 253)	NDN (*n* = 253)	OR (95% CI)	*p*	SD
Sex			1.00 (0.70–1.44)	1.000	0.00%
Male	162(64.0)	162(64.0)			
Female	91(36.0)	91(36.0)			
Age	64.5 ± 12.3	64.5 ± 12.3	―	0.986	0.14%
GCS	13.5 ± 3.1	13.5 ± 3.1	―	0.977	0.07%
SIH vs. DH	SIH (*n* = 178)	DH (*n* = 178)	OR (95% CI)	*p*	SD
Sex			1.00 (0.65–1.55)	1.000	0.00%
Male	117(65.7)	117(65.7)			
Female	61(34.3)	61(34.3)			
Age	62.5 ± 14.3	62.8 ± 13.7	―	0.839	−1.71%
GCS	12.9 ± 3.6	13.0 ± 3.5	―	0.893	−1.12%
Kaohsiung CGMH					
DN vs. NDN	DN (*n* = 352)	NDN (*n* = 352)	OR (95% CI)	*p*	SD
Sex			1.00 (0.74–1.35)	1.000	0.00%
Male	198(56.3)	198(56.3)			
Female	154(43.8)	154(43.8)			
Age	68.3 ± 11.3	68.3 ± 11.2	―	0.989	0.02%
GCS	13.3 ± 3.2	13.3 ± 3.2	―	0.944	0.06%
SIH vs. NDN	SIH (*n* = 496)	NDN (*n* = 496)	OR (95% CI)	*p*	SD
Sex			1.00 (0.77–1.30)	1.000	0.00%
Male	331(66.7)	331(66.7)			
Female	165(33.3)	165(33.3)			
Age	50.2 ± 19.6	50.5 ± 19.0	―	0.838	−1.77%
GCS	8.5 ± 4.9	8.5 ± 4.9	―	0.954	0.05%
DH vs. NDN	DH (*n* = 476)	NDN (*n* = 476)	OR (95% CI)	*p*	SD
Sex			1.11 (0.77–1.29)	1.000	0.00%
Male	274(57.6)	274(57.6)			
Female	202(42.4)	202(42.4)			
Age	62.5 ± 12.9	62.5 ± 13.0	―	0.984	−0.02%
GCS	12.1 ± 4.1	12.1 ± 4.1	―	0.975	−0.07%
SIH vs. DH	SIH (*n* = 301)	DH (*n* = 301)	OR (95% CI)	*p*	SD
Sex			1.00 (0.72–1.38)	1.000	0.00%
Male	177(58.8)	177(58.8)			
Female	124(41.2)	124(41.2)			
Age	59.8 ± 14.4	58.8 ± 13.5	―	0.389	4.78%
GCS	10.4 ± 4.8	10.7 ± 4.6	―	0.362	−5.92%

CGMH = Chang Gung Memorial Hospital; DH = diabetic hyperglycemia; DN = diabetic normoglycemia; NDN = nondiabetic normoglycemia; SIH = stress-induced hyperglycemia; SD: standardized difference.

**Table 4:** Comparison of the mortality outcome among the four groups (NDN, DN, SIH, DH) within each hospital after propensity matching.

Propensity Score-Matched Populations	Mortality, Group: *n* (%)		OR (95% CI)	*p*
Keelung CGMH				
DN (*n* = 56) vs. NDN (*n* = 56)	DN: 2(3.6)	NDN: 2(3.6)	1.00 (0.06–15.99)	1.000
SIH (*n* = 102) vs. NDN (*n* = 102)	SIH: 18(17.6)	NDN: 9(8.8)	3.25 (1.06–9.97)	0.039
DH (*n* = 53) vs. NDN (*n* = 53)	DH: 4(7.5)	NDN: 2(3.8)	3.00 (0.31–28.84)	0.341
SIH (*n* = 47) vs. DH (*n* = 47)	SIH: 6(12.8)	DH: 5(10.6)	1.25 (0.34–4.66)	0.739
Linkou CGMH				
DN (*n* = 279) vs. NDN (*n* = 279)	DN: 15(5.4)	NDN: 12(4.3)	1.33 (0.56–3.16)	0.514
SIH (*n* = 810) vs. NDN (*n* = 810)	SIH: 171(21.1)	NDN: 106(13.1)	2.08 (1.53–2.83)	<0.001
DH (*n* = 499) vs. NDN (*n* = 499)	DH: 44(8.8)	NDN: 50(10.0)	0.83 (0.51–1.35)	0.461
SIH (*n* = 399) vs. DH (*n* = 399)	SIH: 79(19.8)	DH: 39(9.8)	2.82 (1.73–4.58)	<0.001
Chiayi CGMH				
DN (*n* = 180) vs. NDN (*n* = 180)	DN: 5(2.8)	NDN: 4(2.2)	1.25 (0.34–4.66)	0.739
SIH (*n* = 304) vs. NDN (*n* = 304)	SIH: 33(10.9)	NDN: 25(8.2)	1.50 (0.80–2.82)	0.209
DH (*n* = 253) vs. NDN (*n* = 253)	DH: 9(3.6)	NDN: 5(2.0)	2.33 (0.60–9.02)	0.220
SIH (*n* = 178) vs. DH (*n* = 178)	SIH: 14(7.9)	DH: 8(4.5)	1.86 (0.74–4.66)	0.187
Kaohsiung CGMH				
DN (*n* = 352) vs. NDN (*n* = 352)	DN: 12(3.4)	NDN: 13(3.7)	0.91 (0.39–2.14)	0.827
SIH (*n* = 496) vs. NDN (*n* = 496)	SIH: 90(18.1)	NDN: 66(13.3)	1.63 (1.09–2.44)	0.017
DH (*n* = 476) vs. NDN (*n* = 476)	DH: 36(7.6)	NDN: 33(6.9)	1.11 (0.66–1.87)	0.691
SIH (*n* = 301) vs. DH (*n* = 301)	SIH: 51(16.9)	DH: 32(10.6)	1.91 (1.12–3.23)	0.017

CGMH = Chang Gung Memorial Hospital; DH = diabetic hyperglycemia; DN = diabetic normoglycemia; NDN = nondiabetic normoglycemia; SIH = stress-induced hyperglycemia.

## Abbreviations

(CGMH)	Chang Gung Memorial Hospital
(CGRD)	Chang Gung Research Database
(DH)	Diabetic hyperglycemia
(ICD-9)	International Classification of Diseases, Ninth Revision
(DM)	Diabetes mellitus
(DN)	Diabetic normoglycemia
(HbA1c)	Glycated hemoglobin
(NDN)	Nondiabetic normoglycemia
(SIH)	Stress-induced hyperglycemia
(TBI)	Traumatic brain injury
